# p53 Dependent Apoptotic Cell Death Induces Embryonic Malformation in *Carassius auratus* under Chronic Hypoxia

**DOI:** 10.1371/journal.pone.0102650

**Published:** 2014-07-28

**Authors:** Paramita Banerjee Sawant, Aritra Bera, Subrata Dasgupta, Bhawesh T. Sawant, Narinder K. Chadha, Asim K. Pal

**Affiliations:** 1 Central Institute of Fisheries Education (ICAR), Versova, Mumbai, Maharastra, India; 2 Taraporewala Marine Biological Research Station, (Konkan Krishi Vidyapeeth), Mumbai, Maharastra, India; University of Hawaii Cancer Center, United States of America

## Abstract

Hypoxia is a global phenomenon affecting recruitment as well as the embryonic development of aquatic fauna. The present study depicts hypoxia induced disruption of the intrinsic pathway of programmed cell death (PCD), leading to embryonic malformation in the goldfish, *Carrasius auratus*. Constant hypoxia induced the early expression of pro-apoptotic/tumor suppressor p53 and concomitant expression of the cell death molecule, caspase-3, leading to high level of DNA damage and cell death in hypoxic embryos, as compared to normoxic ones. As a result, the former showed delayed 4 and 64 celled stages and a delay in appearance of epiboly stage. Expression of p53 efficiently switched off expression of the anti-apoptotic Bcl-2 during the initial 12 hours post fertilization (hpf) and caused embryonic cell death. However, after 12 hours, simultaneous downregulation of p53 and Caspase-3 and exponential increase of Bcl-2, caused uncontrolled cell proliferation and prevented essential programmed cell death (PCD), ultimately resulting in significant (p<0.05) embryonic malformation up to 144 hpf. Evidences suggest that uncontrolled cell proliferation after 12 hpf may have been due to downregulation of p53 abundance, which in turn has an influence on upregulation of anti-apoptotic Bcl-2. Therefore, we have been able to show for the first time and propose that hypoxia induced downregulation of p53 beyond 12 hpf, disrupts PCD and leads to failure in normal differentiation, causing malformation in gold fish embryos.

## Introduction

Hypoxia (<2.8 mg dissolved oxygen (DO) / litre water), is an inevitable problem in aquatic ecosystems worldwide and is likely to be exacerbated by climate change in the coming years. Hypoxic dead zones in large estuaries and marine systems are a worldwide phenomenon and generally occur in river outflows due to organic run-off and corresponding eutrophication [1], [2]. Study on effects of environmental factors on biological systems has become a central issue in developmental biology [3] and oxygen, being the final electron acceptor during oxidative phosphorylation, is the first absolute requirement for all life forms. This makes oxygen an important metabolic regulator during vertebrate embryogenesis. The rate of diffusion of oxygen in water is only 1/10000^th^ that of air and water contains 1/30^th^ of the oxygen contained in the same volume of air at the same partial pressure [4], [5]. Since water has low oxygen solubility, most fish species have evolved behavioral, anatomical, physiological, biochemical and molecular adaptations that enable them to cope with periods of hypoxia [6], [7], [8]. Hypoxia has been revealed to cause significant morphological and gene transcriptional changes in adult fish [9], [10], [11], [12], [5], [13]. However, the molecular and biochemical mechanisms of hypoxia tolerance in fish embryos remain largely unclear [14]. It is generally accepted that embryonic and larval developmental stages are much more sensitive to environmental challenges than adults and this becomes more critical during embryonic development in case of rapidly growing cells, when exposed to hypoxia [15]. While aquatic hypoxia acts as a teratogen and causes malformation in zebrafish embryos, delayed development has been also reported in embryos of mussel and black bream, developmental defects in grass carp embryos, male dominated population in zebrafish, yolk sac fry deformity and vertebrate column deformity of salmon [15], [16], [4], [1], [5], [17]. During normal embryonic development, excess cells are commonly removed by apoptosis, which is an essential mechanism for normal tissue remodeling and morphogenesis during the very delicate phase of embryogenesis. Apoptosis is a well documented part of normal development in a number of tissues. For example, embryonic cavity is formed by apoptosis of epiblast cells in a developing mouse embryo. Apoptotic cells are also present in the notochord of frog (*Xenopus* sp.) embryos, 14 hpf zebrafish and chick embryos [18]. Hence, disruption in apoptosis and change in apoptotic pattern may lead to subsequent malformation. Such changes in the pattern of apoptosis during the development of fish embryos may be caused by hypoxia [15]. Embryonic cells of gold fish at blastula stage induce 211 hypoxic genes under hypoxia and majority of the transcriptional responses to hypoxia are mediated by the hypoxia- inducible factor (HIF-1α) [14].

Previous studies have demonstrated that hypoxic treatment produces an increased expression of both pro and anti-apoptotic proteins such as p53 and Bcl-2, Caspase-9, Caspase-3 respectively at earlier stages of vertebrate embryonic development [19], [20], [21]. In *Xenopus*, this apoptotic cell death is prevented by over-expression of the anti-apoptotic factor, Bcl-2, suggesting that apoptosis in embryos occur through the mitochondrial pathway [18]. The tumor suppressor gene, p53 is one of most frequently mutated genes identified in various types of cancer. Its product, a 53-kDa nucleophosphoprotein is a multifunctional protein that plays pivotal regulatory role in cell cycle checkpoints, genetic stability, apoptosis and DNA repair. Human p53 has five domains and most of the mutations that deactivate p53 occur in central DNA binding domain (DBD). The two conserved functions of mammalian p53, namely, tumor suppression through maintenance of genomic integrity and induction of apoptosis have also been established for fish p53 [19]. Several apoptosis related genes that are transcriptionally regulated by p53 have been identified. Besides the zebrafish (*Danio rerio*), p53 gene sequences have been reported from barbel (*Barbus barbus*), flounder (*Platichthys flesus*), medaka (*Oryzias latipes*), pufferfish (*Fugu rubripes*), platy (*Xiphophorus maculates*), pufferfish (*Tetraodon miurus*), and rainbow trout (*Oncorhynchus mykiss*) [19]. Further, p53 induction in fish can be used as biomarker of exposure to genotoxic chemicals [19], as it can also be induced in fish under DNA damage or hypoxia. [22].

In this study, we have used goldfish embryos to investigate how hypoxia affects embryonic development. The goldfish is an excellent model for this study because the stages of its embryonic development have been well documented, which permitted us to map its pattern of development under hypoxia. Here, we have provided evidence that goldfish embryos showed malformation during hypoxia exposure, via p53 dependent apoptotic pathway up to 144 hpf and we have also been able to examine the role of apoptosis and cell proliferation in relation to embryonic malformation. Our results are deemed to provide significant understanding of the adaptive mechanism by which goldfish embryos respond to hypoxia.

## Materials and Methods

### Goldfish maintenance and embryo collection

Thirty pairs of broodstock of *C. auratus* males (55±0.05 g) and females (46±0.05 g) were reared and maintained according to conditions best suited for breeding goldfishes. Fishes were kept at 22°C in aerated water (6.5 mg/l DO) and subjected to a 14L: 10D cycle of photoperiod for 2 weeks during the month of December. Broodstock were induced bred using Ovatide (HemmoPharma, Mumbai, India) @ 0.3 ml/kg (females) and 0.2 ml/kg (males) respectively. Subsequently, spawners were maintained in separate glass chambers and stripped after 12 h. Eggs and milt were collected by gently stripping the spawners into clean and dry porcelain vials and pooled to ensure genetic variability. Pooled eggs and milt were mixed gently by a feather to ensure effective fertilization in each tray. Fertilized eggs were hardened by mixing with clean and aerated fresh water at an ambient temperature of 28°C.

Fertilized eggs were selected and instantly transferred into hypoxic and normoxic chambers containing filtered freshwater and incubated at 28°C. The developmental stages of embryos were described as hours post fertilization (hpf) and classified according to their morphological characteristics described in earlier studies. [23], [24]

### Ethics Statement

The research undertaken complies with the current animal welfare laws in India. Even though fish embryos have been used in the present study, no animals have been stressed or sacrificed for the same. However, care and treatment of brood stock used in this study for procurement of eggs, were in accordance with the guidelines of the CPCSEA [(Committee for the Purpose of Control and Supervision of Experiments on Animals), Ministry of Environment & Forests (Animal Welfare Division), Govt. of India] on care and use of animals in scientific research. The study was undertaken with approval of statutory authorities of the Central Institute of Fisheries Education, Mumbai, India (University under Sec. 3 of University Grants Commission Act and ISO 9001∶2008 certified). As the experimental fish, *Carassius auratus* is not an endangered fish, the provisions of the Govt. of India's Wildlife Protection Act of 1972 are not applicable for experiments on this fish.

### Controlled atmospheric chambers

Embryos were randomly distributed into cylindrical glass chambers containing 3 liters of filtered fresh water at two different concentrations of DO in the laboratory, with an ambient temperature of 28°C. Treatments consisted of 6 replicates with each replicate containing 3300±5.2 eggs, so that the total numbers of eggs stripped were approximately 20,000, from 8 female goldfishes, considering average fecundity of an adult female of *C. auratus* to be 2500.

Initial viability assessment was conducted using 550±1.2 eggs, distributed in 12 glass chambers [one normoxic (6.5 mg/l DO) and three hypoxic (0.5, 1, and 2 mg/l DO)], each having three replicates with each replicate containing 46 numbers of eggs. The levels of DO (mentioned above), were maintained using a digitized automated cut off system of air flow and nitrogen gas into water kept in the glass chambers.

### Viability assay

A preliminary study [15] was conducted to determine the suitable and approximate sub lethal level of DO, in relation to viability of goldfish embryos. This was accomplished by conducting a cumulative death study (CDS) of the embryos under four levels of dissolved oxygen, (6, 2, 1 and 0.5 mg/l DO). Embryogenesis of gold fish is generally completed within 72 h and development of important internal organs takes place within 24, 48, 72, 96, 120 and 144 h respectively [23], [24], [15]. CDS conducted during the above mentioned time intervals revealed that 69% of embryos are able to survive and develop up to 144 h on exposure to 1 mg/l DO. Taking cue from the above, embryos were exposed to 1 mg/l DO (hypoxia) and 6 mg/l DO (normoxia) for further experimentation.

### Malformation and body length assessment

Body length was measured and malformation assessed [15] using a trinocular microscope with an attached CCD camera and calibrated scale respectively. Spinal malformations were counted for estimation of percentage of malformation under hypoxia and normoxia.

### Somite numbers

Somite numbers of embryos were counted under a dissecting microscope in normoxic and hypoxic groups at 12 h, 24 h, 34 h, 44 h and 72 h respectively and each stage was determined according to a previous study [23].

### Preparation of single cell suspension

Single cell suspension of embryos were prepared [25] using HEPES buffer, HEPES Collagenase D solution, DNase-I, PBS and PEB buffers.

### Characterisation of pattern of cell death and cellular DNA fragmentation

Cellular DNA fragmentation was assayed using cellular DNA fragmentation ELISA kit (Roche Biosciences, Germany), which uses photometric ELISA for detection of BrdU labelled DNA fragments in cell lysates or cell culture supernatants. BrdU is used as a metabolic labeling agent by the nuclear DNA of target cells. In this case, cells were adjusted to 2-4×10^5^ cells per ml of culture medium and incubated for 2 h at 37°C, for labeling with BrdU solution, according to manufacturer's instructions. This assay has been used in the present study to ascertain whether apoptotic cell death (by detection of BrdU labeled DNA fragment in the cytoplasm of affected cells in the cell lysates) or necrotic cell death (detection of BrdU labeled DNA fragments in the supernatants of cell lysates) has taken place during 0.75 h and 2 h (cleavage), 4 h (Blastula), 7 h (Gastrula), 12 h (Somite) and 24 h (Pharyngula) stages of embryonic development.

### Caspase-3 *assay*

Embryonic single cell preparation was adjusted to 1×10^7^ cells/ml of the culture medium and Caspase 3 ELISA assay was performed for embryos at 0.75 h and 2 h (cleavage), 4 h (Blastula), 7 h (Gastrula), 12 h (Somite) and 24 h (Pharyngula) stages respectively, according to manufacturer's instructions from Sigma Aldrich, USA.

### Cell proliferation assay

Cell proliferation assay was performed during 0.75 h and 2 h (cleavage), 4 h (Blastula), 7 h (Gastrula), 12 h (Somite) and 24 h (Pharyngula) stages respectively, during which, embryonic single cell preparation was adjusted to 0.1-1×10^5^ cells/ml of culture medium for labeling with BrdU solution. The reaction incorporates the pyrimidine analogue of BrdU in place of thymidine into the DNA of proliferating cells. The anti-BrdU peroxide (POD) then binds to the BrdU incorporated into newly synthesized cellular DNA. The immune complexes thus formed were detected by the subsequent substrate reaction. The reaction product was quantified by measuring the absorbance at A370 nm – A492 nm, by ELISA, according to manufacturer's instruction (Roche Biosciences, Germany) and absorbance values directly correlated to the amount of DNA synthesis and to the number of proliferating cells in the respective wells of the micro-culture.

### p53 and Bcl-2 assay

p53 and Bcl-2 assay were performed during 0.75 h and 2 h (cleavage), 4 h (Blastula), 7 h (Gastrula), 12 h (Somite) and 24 h (Pharyngula) stages according to manufacturer's instructions (Invitrogen, USA), with adjusted cell suspension of 5×10^6^ cells per ml. In this assay, p53 and Bcl-2 sensitivity were recorded as 0.33 U/ml and <0.5 ng/ml respectively.

### Statistical Analysis

Paired‘t’ test and one way ANOVA were used to test the possible significant differences between the mean of each parameter measured in normoxic and hypoxic groups respectively, using SPSS ver. 19.0. Differences were considered significant if *p*<0.05.

## Results

### Viability assay

**Figure 1:**
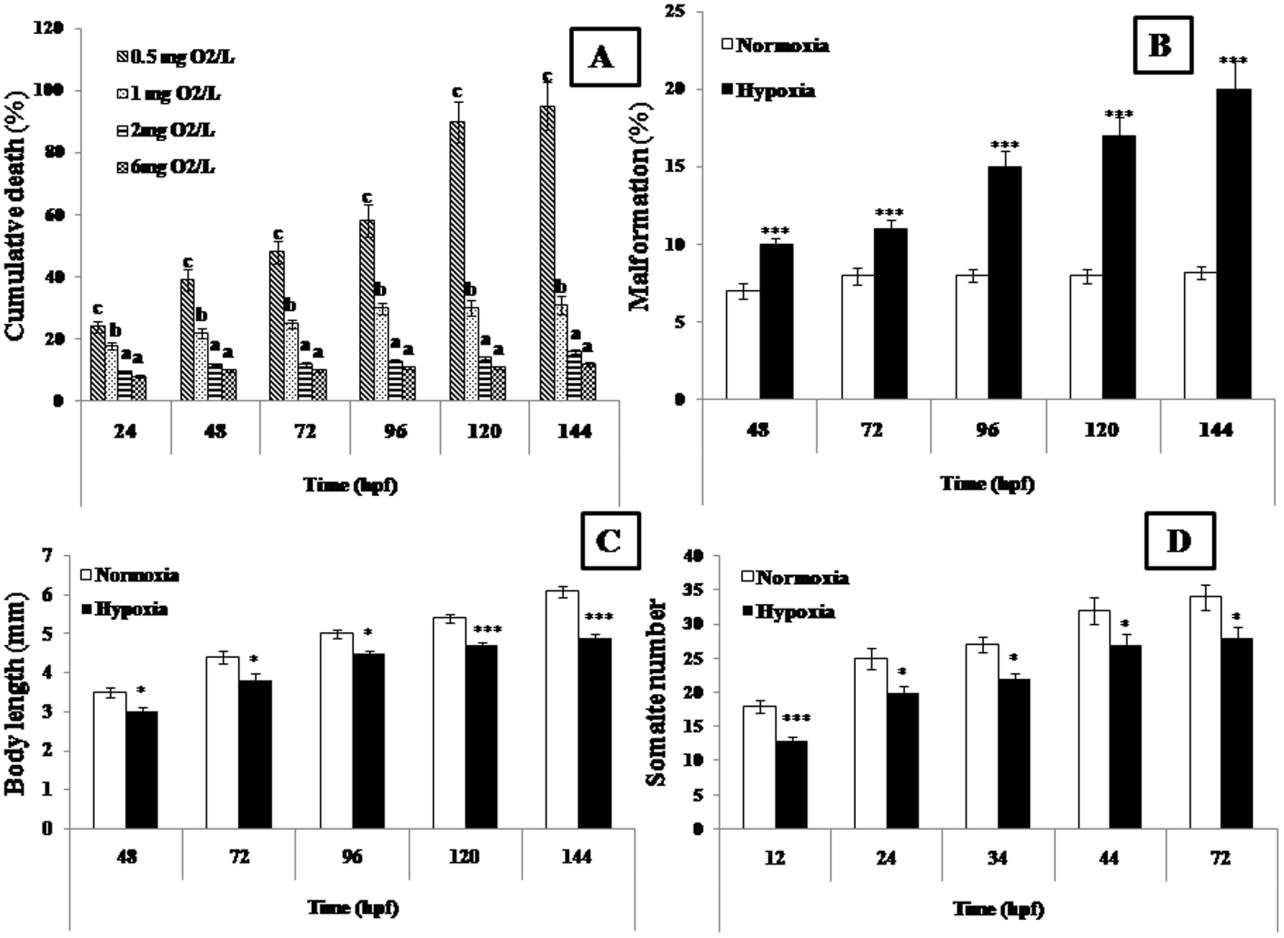
Malformation in gold fish embryos under normoxia and hypoxia. (A). Viability assay (% of cumulative death): Gold fish embryos showed 100% mortality under anoxia >15 hours and were thus found to be anoxia intolerant. After 144 h, percentages of dead embryos were 95%, 31%, 16% and 12 % in 0.05, 1, 2, and 6 mg/l DO respectively. (B). Malformation (%): After 144 h, percentage of embryos with malformation in hypoxia group was significantly higher (p<0.001) than that of normoxia. (C). Body length: After 144 h, embryos exposed to hypoxia showed significantly (p<0.001) shorter body length than that of control group. (D). Somite Numbers: Highest somite reduction was observed during 12 hpf compared to control. Different superscripts in cumulative death assay indicate significant differences (p<0.05) amongst different treatments and control. Asterisks indicate significant differences between normoxic and hypoxic groups (paired t test, *p<0.05 and *** *p*<0.001) at different times. Values are expressed as mean ± SEM (n = 20).

Gold fish embryos were found to be anoxia intolerant as they showed 100% mortality under anoxia for more than 15 hours (Fig. 1A). Concentration of oxygen and mortality of gold fish embryos showed a positive correlation. After 144 h, percentages of dead embryos were 95%, 31%, 16% and 12 % in 0.05, 1, 2, and 6 mg/l DO respectively. Highest (*p*<0.05) mortality (95±7.6 %) was observed in 0.5 mg/l DO hypoxic group whereas control, 2 mg/l DO and 1 mg/l DO groups recorded 12 %, 16% and 31% mortality respectively. There were no significant differences in viability (*p*>0.05) between normoxia and 2 mg/l DO groups.

### Kinetics of embryonic development and malformation

**Figure 2:**
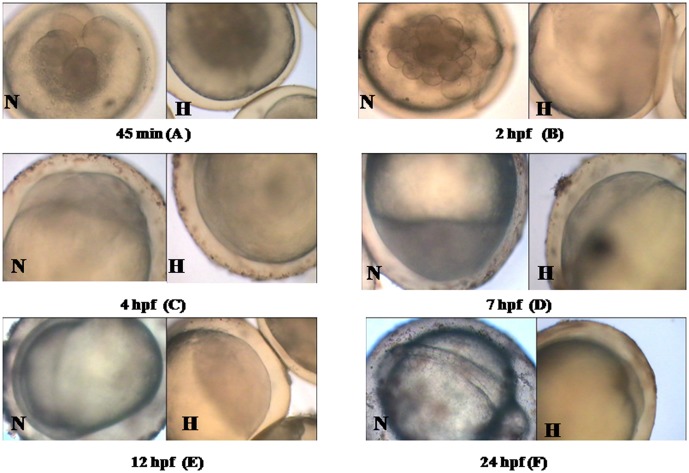
Embryonic development of Goldfish under normoxia and hypoxia. (A) Embryos at 0.75 hpf (normoxia) showing blastodisc with four blastomeres, after appearance of the second cleavage furrow (vertical meridional) under hypoxia showing delayed 4 cell stage compared to the normoxia counterpart. (B). Embryos at 2 hpf: Embryos (normoxia) showing normal blastoderm with a composed mass of relatively small but distinct cells: under hypoxia showing delayed 64 celled stage, compared to the normoxia counterpart. (C). Embryos at 4 hpf: Epiboly stage arrived in normoxic embryos showing multicellular blastoderm, margin of which extends slightly over yolk. (D). Embryos at 7 hpf: Epiboly stage was delayed by 3 hours in hypoxic embryos. (E). Embryos at 12 hpf: Appearance of shield stage in normoxic embryos showing its visible axis with a narrow, transparent ridge, extending forward from the blastopore, encircling the yolk. The same was not observed in hypoxic counterparts. (F). Embryos at 24 hpf: Normoxic embryos showing somite stage whereas hypoxic embryos reaching shield stage within the same time, signifying delayed development by approximately 16 hrs. Scale: 200 µm.

Hypoxia caused delayed development in gold fish embryos. Embryos exposed to 1.0 mg/l DO showed significant delay in embryogenesis compared to normoxia (Fig. 2 A-F) Such delay in developmental process was initially observed at 4 celled stage in hypoxic embryos compared to their normoxic counterpart. Epiboly stage was delayed by 3 h in hypoxic embryos. After 24 h exposure to hypoxia, embryos reached shield stage whereas at the same time, normoxic embryo had reached somite stage and development was delayed by approximately 16 h. As a result, after 144 h, the average body length (paired t test *p*<0.001) of embryos in hypoxic group were significantly (paired t test *p*<0.001) shorter (4.9±0.11 mm) than that of control (normoxic) group (6.1±0.14 mm) (Fig. 1C).Percentage of malformation (Fig. 1B) was measured by external abnormalities like spinal deformities. After 144 h, percentage of hatchlings with malformation (20±1.83 %) in hypoxia group was significantly higher (paired t test *p*<0.001) than that of normoxia (8.2±0.4 %). Although similar trend were observed at 48, 72, 96 and 120 h respectively, percentage of malformation was highest at 144 h.

### Somite number

After 12 h of hypoxia, there were significant differences (*p*<0.05) in somite numbers among the hypoxic groups at 24 hpf, 34 hpf, 44 hpf and 72 hpf respectively. Highest somite reduction (13±0.59) was observed at 12 hpf compared to control (normoxia) (18±0.90) (Fig. 1D).

### Characterization of type of cell death

**Figure 3:**
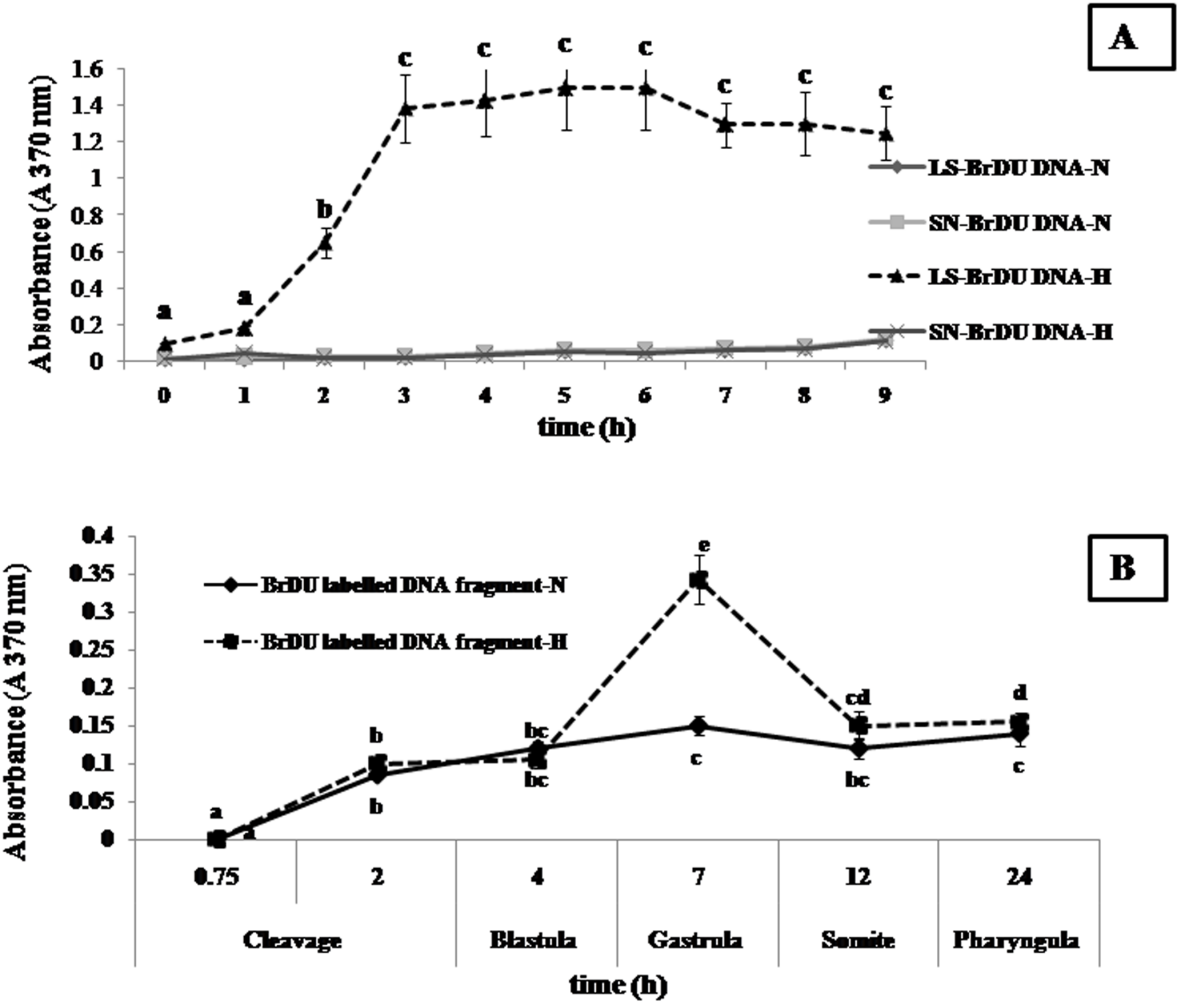
Characterization of pattern of cell death and cellular DNA fragmentation. (A). Type of cell death (Apoptotic or Necrotic): DNA fragments in hypoxic cell lysate significantly increased (*p*<0.05) after 1 h of incubation and reached highest level at 3 h and continued to be high up to 9 h, with a small peak at 6 h. No BrdU labeled DNA fragment was detected in the hypoxic cell supernatants during first 4 hours of incubation, indicating that DNA fragmentation had occurred prior to lysis of plasma membrane; Hence, cause of cell death may be attributed to apoptosis and not due to necrosis, under hypoxia. (B). DNA fragmentation: Phenomena of cellular DNA fragmentation under hypoxia started increasing after blastula (4 hpf) stage and reached its highest (*p*<0.05) during gastrula phase (7 hpf), thereafter returning to the level of control after 12 h. Different superscripts indicate significant differences (p<0.05) amongst normoxic and hypoxic groups at different times. Values are expressed as mean ± SEM (n = 10).

The kinetics of incubation of pre exposed 1×10^4^ embryonic cell suspensions (after pre exposure for 45 min in hypoxic media), showed DNA fragments appearing first in the cell lysates. DNA fragments in cell lysates significantly increased (*p*<0.05) after 1 h of incubation, reached its highest level at 3 h and thereafter, remained at a similar level. No BrdU labeled DNA fragment was detected in the cell supernatants during the first 4 h of incubation, indicating that DNA fragmentation occurred prior to lysis of plasma membrane. Therefore, cell death was caused due to apoptosis in this case since necrotic cells generally release DNA fragments into supernatants at a very early stage (<4 h) of cell death (Fig. 3A).

### Cellular DNA fragmentation

Rate of cellular DNA fragmentation started increasing after blastula (4 hpf) stage and reached its highest (*p*<0.05) during gastrula phase (7 hpf) thereafter declining to normal,l as shown by control group after 12 h (somite stage) (Fig. 3B).

### Caspase-3

**Figure 4:**
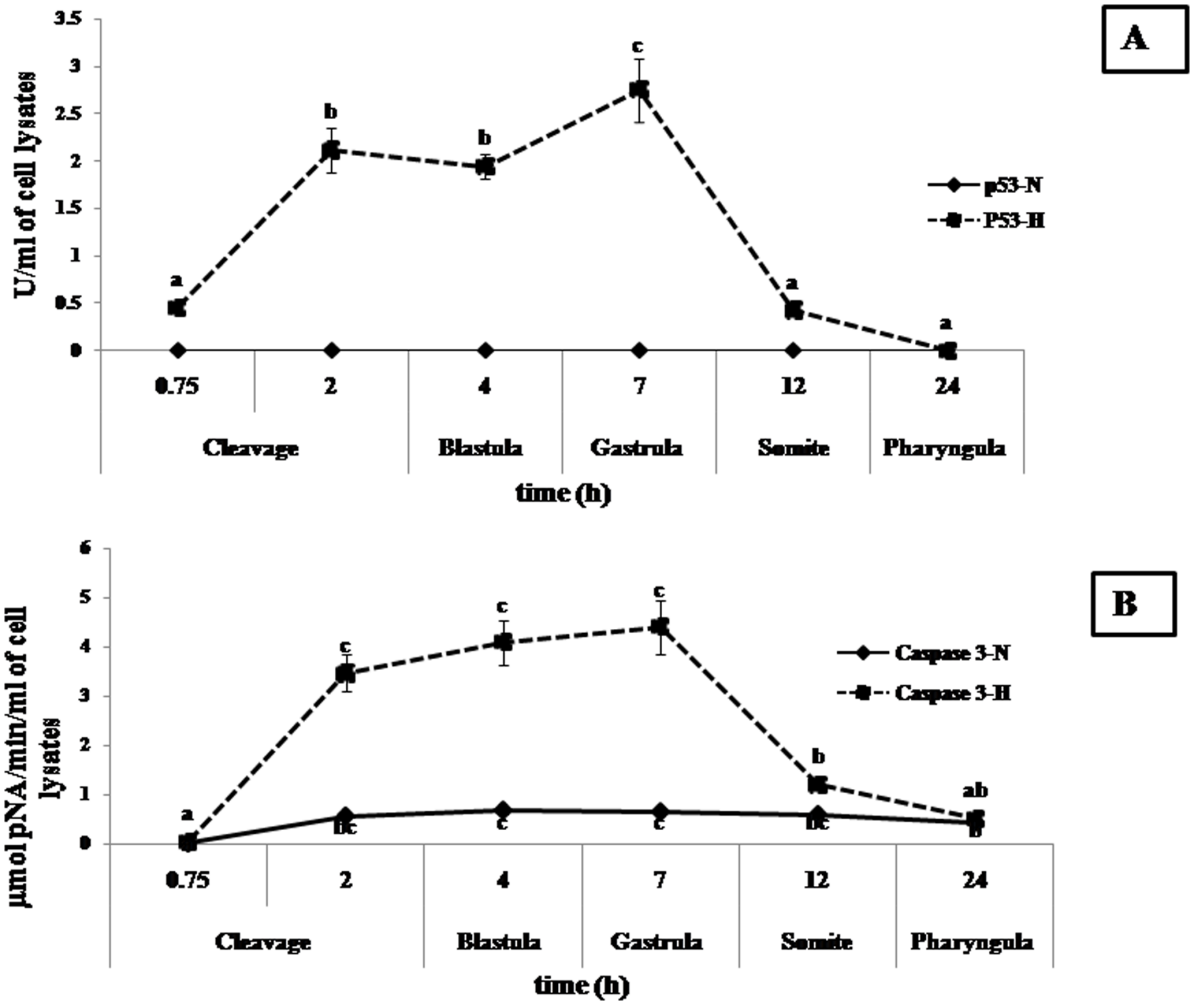
p53 and Caspase-3 assay of embryonic cell under normoxia and hypoxia. (A). p*53*: p*53* expression was highest (*p*<0.05) during 7 hpf, Beyond 24 h, p53 did not show any expression. p53 expression was not detected in normoxic embryos,. (B). Caspase 3: Caspase-3 showed significantly higher (p<0.05) expression in early stages during 2 hpf (cleavage stage) in hypoxia exposed embryos compared to control (normoxia). Different superscripts indicate significant differences (*p*<0.05) amongst hypoxic and normoxic groups at different times. Values are expressed as mean ± SEM (n = 10).

Caspase-3 showed significantly higher (*p*<0.05) expression during 2 hpf (cleavage stage) at a level of 3.46±0.37 µmol pNA/min/ml of cell lysate in hypoxic embryos compared to control (normoxic). Higher levels of caspase-3 thus recorded at 2 hpf, decreased to levels similar to control during 12 hpf. Thereafter, the level of Caspase 3 showed significant increase (*p*<0.05) in normoxic embryos from 2 hpf to 12 hpf, although level of expression was too low (0.55±0.05, 0.67±0.07, 0.64±0.07 and 0.58±0.04 µmol pNA/min/ml of cell lysate) compared to its hypoxic counterpart (3.46±0.37, 4.09±0.45, 4.40±0.55 and 1.20±0.11 µmol pNA/min/ml of cell lysate, during 2, 4, 7 and 12 hpf respectively) (Fig. 4B).

### Cell Proliferation

**Figure 5:**
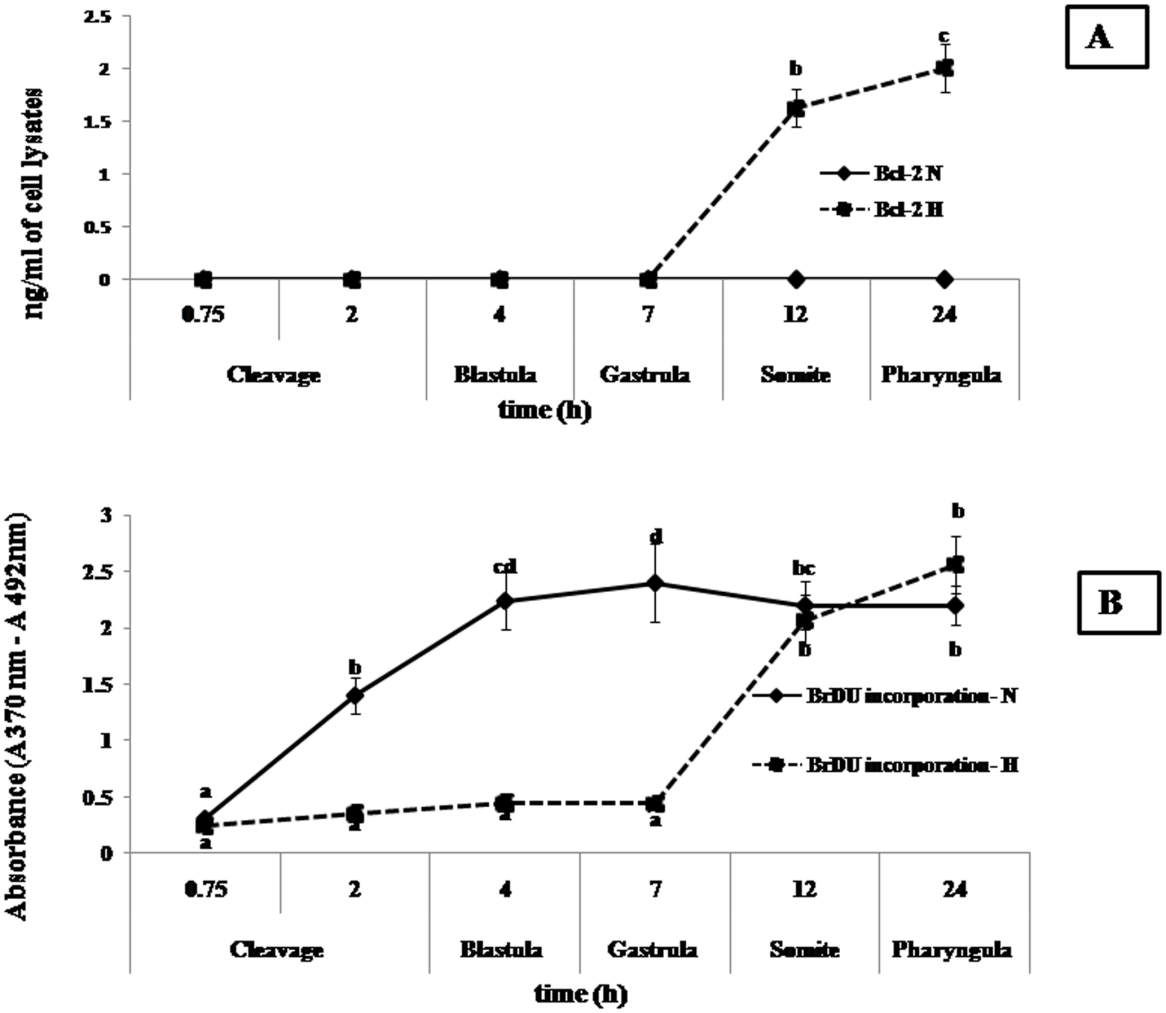
Bcl-2 and cell proliferation assay of embryonic cells under normoxia and hypoxia. (A). Bcl-2 assay: Bcl-2 reached its highest (p<0.05) level in cell lysates during 24 hpf. Bcl-2 expression was not detected in normoxic embryos during embryonic development. (B).Embryonic cell proliferation: Normoxic embryos showed continuous cell proliferation till 24 hpf, whereas hypoxic embryos showed no significant cell proliferation up to 7 hpf (gastrula phase), followed by abrupt increase in proliferation after 7 hpf up to 24 hpf. Different superscripts indicate significant difference (*p*<0.05) amongst normoxic and hypoxic groups at different times. Values are expressed as mean ± SEM (n = 10).

Normoxic embryos showed continuous normal cell proliferation till 24 hpf,, which was not the case in the hypoxic group.. The latter (hypoxic group) did not exhibit significant (*p*<0.05) cell proliferation upto 7 hpf (gastrula phase).Thereafter, hypoxic embryos showedan abrupt increase in cell proliferation (*p*<0.05) after 7 hpf through 24 hpf (signified by absorbance values, 0.44±0.05, 2.07±0.21, 2.24±0.25 respectively, measured at wavelength A370 nm–492 nm during 7, 12 and 24 hpf respectively), and significantly exceeded that of control (normoxic group) 12 hpf onwards, as evidenced by higher level of incorporation of the pyrimidine analogue of BrDU in place of thymidine into the DNA of proliferating cells. The anti-BrdU peroxide (POD) bound to the BrdU incorporated into the newly synthesized cellular DNA have been detected by the subsequent substrate reaction. The reaction product quantified and absorbance values directly correlated to the amount of DNA synthesis and to the number of proliferating cells in the respective wells of the micro-culture. (Fig. 5B).

### p53 *and* Bcl-2

Expression pattern of the apoptotic protein p53, in hypoxic embryos was similar to that of caspase-3. Expression of p53 was highest (*p*<0.05) during 7 hpf at a level of 2.75±0.33 U/mol of cell lysates. Beyond 24 h, p53 did not show any expression. p53 expression was not detected in normoxic embryos (Fig. 4A).

Anti-apoptotic Bcl-2 expression pattern was opposite to that of p53. Bcl-2 reached its highest (*p*<0.05) level during 24 hpf at 2.01±0.23 ng/ml of cell lysate. There was no Bcl-2 expression in hypoxic embryos up to 7 hpf and at a time when p53 expression was the highest. Bcl-2 expression was not detected in normoxic embryos (Fig. 5A).

## Discussion

Hypoxia is an important environmental stressor that has manifold effects on aquatic life. Earlier studies have demonstrated [16] that embryonic developmental stages are much more sensitive to environmental challenges than adult stages. Embryonic developmental stages comprising of embryonic histogenesis and organogenesis are highly dependent on programmed cell death (PCD) via the apoptotic pathway. Hypoxia can potentially alter normal apoptotic pattern in vertebrate embryonic development leading to malformation and teratogenic effect [15]. For the first time, we reported the probable mechanism of malformation due to the disruption of the PCD infrastructure, via the apoptotic p53 and anti-apoptotic Bcl-2, leading to teratogenic effect in embryonic stages of a hypoxia tolerant cyprinid, the goldfish (*Carassius auratus*). The study reveals that the disturbance in cell proliferation may be attributed to such malformation and demonstrated for the first time that, the expression and inhibition of p53 and Bcl-2 during different phases of development were responsible for uncontrolled cell proliferation.

### Embryonic malformation under hypoxia

Hypoxia can cause delay in embryonic development, mortality, teratogenic effect and spinal deformity in fish by altering normal apoptotic patterns during development [15], [16], [17], [21]. Severe hypoxic conditions caused high mortality and impaired hatching in black bream, during the first day of embryonic development. Further, severely hypoxic conditions resulted in abnormal development in all black bream embryos, resulting in an undifferentiated mass of cells towards the animal pole and a lack of cephalization. Therefore, even though hypoxia, either *in vivo* or *in vitro* is capable of inducing apoptosis in various cell types, the pattern of apoptosis becomes essentially different during hypoxic conditions. In the present study, goldfish embryos proved to be anoxia intolerant and showed 100 % mortality during the first 24 h, whereas 69 % survival was recorded after 144 h of hypoxia exposure (1 mg/l DO), similar to earlier studies on zebrafish (*Danio rerio*) [15]. Additionally, high percentage of malformation was observed in gold fish embryos in the present study. Causes of malformation may be attributed to delayed embryonic development leading to significant decrease in somite number and retarded body length of embryos, alike zebrafish embryos [15].

### Hypoxia triggered p53 and caused apoptosis

The principal function of p53 is to promote survival or deletion of cells exposed to agents that cause DNA damage, such as hypoxia, UV radiation or reactive oxygen species in fish [26]. Its role as a biomarker has also been suggested [26]. The p53 tumor suppressor gene limits cellular proliferation by inducing cell cycle arrest and apoptosis in response to cellular stresses such as hypoxia and DNA damage. Many apoptosis-related genes that are transcriptionally regulated by p53 have been identified as potential candidates for implementing p53 effector functions [22]. In hypoxic goldfish embryos, p53 showed its highest expression during first 12 hpf (Fig. 4 A), which can be signified as the phase of prolific apoptotic cell death and DNA damage. p53 gene was also transiently expressed during signal transduction followed by rapid decrease in medaka [27]. In earlier studies, microarray of blastulae embryonic cells using suppression subtractive hybridization in gold fish embryos under hypoxia showed p53 gene expression [14]. Similar to hypoxia, exposure of the hermaphroditic fish, *Kryptolebias marmoratus* to endocrine disrupting chemicals (EDCs) such as bisphenol A, 4-nonylphenol, and 4-tert-octylphenol also triggers expression of p53 gene at different stages of embryonic development. p53 gene expression could be detected at 4 day post fertilization (dpf) in embryos of *K. marmoratus*, exposed to EDCs. The expression was p53 upregulated at 9 dpf and downregulated thereafter [28]. We also found a similar pattern of upregulation in expression p53 at 12 hpf, followed by its gradual decrease/downregulation. Since hypoxia acts as an endocrine disrupter in adult and embryonic stages of fishes much like certain chemicals, such as EDC, [3], [15], [1] similarity in their action pathway in up-regulation of p53 may not be ignored. Generally, there are two pathways for apoptosis *i.e.* intrinsic and extrinsic. The intrinsic pathway of apoptosis is activated in chronic hypoxia which includes both pro and anti-apoptotic members of the Bcl-2 family. Activation of the hypoxia induced intrinsic apoptotic pathway leads to up-regulation of p53 protein levels, which further leads to the activation of the pro-apoptotic proteins, *p53 Upregulated Mediator of Apoptosis* or Puma. Puma induces expression (via direct transcriptional activation) of the *puma* gene by p53 (as reported in zebrafish embryos, *in vitro* and *in vivo*) [29], resulting in neutralization of the anti-apoptotic Bcl-2 proteins. This leads to oligomerization of the pro apoptotic *Bak* and *Bax* genes and release of cytochrome C from the mitochondria. In resting cells, the activity of *Bax* and *Bak* is blocked by anti-apoptotic Bcl-2 family and Mcl-1. Cytoplasmic cytochrome C activates the Apaf-1 complex (*Apoptotic protease-activating factor-1*), which converts activator pro-caspases (such as Caspase 9) into their mature form. This is an activator Caspase, which activates executioner pro-Caspases (such as Caspases-3 and 7) and induces proteolysis of ICAD (Inhibitor of Caspase-Activated Deoxyribonuclease), the latter being the inhibitor of CAD (Caspase-Activated Deoxyribonuclease) to execute the cell death program. Free CAD moves into the nucleus of the growing embryo, where it cleaves DNA and causes DNA damage, thereafter starting the same p53 dependent programmed cell death cycle (PCD), which has been recorded upto 12 hpf in the present study [29]. This can be correlated to our results in a way that the delayed 4 and 64 celled stage and a 3 hour delay in appearance of epiboly stage in goldfish embryos under hypoxia, possibly suggests the frequent reprogramming of the p53 machinery resulting in cell death and DNA damage.

### Caspase-3 mediated apoptotic cell death and DNA fragmentation

Programmed cell death (PCD) is an important process in the cellular cycle during tissue development in vertebrates and invertebrates and apoptosis is a physiological mechanism of cell loss [30], [31]. Besides necrosis, apoptotic cell death via caspase-3 is one of the important pathways for PCD. The detection of BrdU labeled DNA fragments in cell lysate helped us to confirm the process of apoptotic cell death in goldfish embryos under hypoxia in the present study, as DNA fragments generally appear in the supernatant of cell cultures at very early stages in case of necrotic types of cell death. Further, in the present study, caspase-3 showed significantly increased activity during the initial 12 hpf, contributing to cellular DNA fragmentation through deactivation of ICAD and activation of CAD [29], which leads cell death, finally resulting in a delay in embryonic development [15]. Therefore, apoptosis resulted under hypoxia through the mitochondrial pathway and continued via p53 dependent apoptosis through activation of Puma gene downstream [18], [29]. Hypoxia must have altered the highly regulated pattern of cell proliferation and apoptosis in embryonic cells by abruptly skewing caspase-3 activity during gold fish embryogenesis. Both apoptotic pathways (intrinsic and extrinsic), converge downstream upon activation of caspases and possibly coordinate the cell dismantling machinery [20].

### Bcl-2 and uncontrolled cell proliferation

Apoptosis is controlled by the Bcl-2 family of proteins, which are generally grouped under pro-apoptotic (Bax/Bad protein) and anti-apoptotic (Bcl-2, Bcl-XL, Bcl-W) proteins and the ratio between these two categories ultimately determines fate of a cell [20], [32]. The present study revealed a significantly higher (above normal) value of the anti-apoptotic Bcl-2 protein in embryos after 12 hpf, indicating uncontrolled cell proliferation, resulting in malformation (20±1.83 % at 144 hpf), due to the reason that the growing embryo is unable to support extra cells during organogenesis, just when p53 dependent apoptosis has been down-regulated by Bcl-2, thus altering the intricate balance of apoptosis and cell proliferation. This occurrence of uncontrolled cell proliferation during vital organogenesis may have possibly resulted in teratogenesis as in case of zebrafish and vertebral column deformity (VCD) in case of salmon under the effect of hypoxia [15], [17]. In the present study, therefore, we have been able to show *prima facia* that, embryonic malformation in cases of chronic hypoxia such as this, results from the disruption of the PCD infrastructure, via apoptotic p53 and anti-apoptotic Bcl-2 in the otherwise hypoxia tolerant cyprinid, *Carassius auratus*,. C1q-like is a novel regulator of cell survival during zebrafish embryogenesis [33]. C1q-like complement proteins might be playing an anti-apoptotic and protective role in inhibiting p53-dependent and caspase 3/9-mediated apoptosis during embryogenesis, since we have hypothesized that early apoptosis must have induced further Bcl-2 dependent cell proliferation which is otherwise termed as apoptosis induced compensatory proliferation (CP), [34] and this pathway has been primarily responsible for malformation in the present case. CP also occurs in other organisms, including *Hydra, Drosophila, Xenopus, Planaria*, newts, and mice. Earlier studies on CP illustrate that both initiator and executioner caspases influence the release of mitogens (chemical substance that encourage cells to commence cell division, thus triggering mitosis) from stressed or injured cells, thereby promoting regeneration, maybe through *Wnt* signaling pathway. Further investigations on this pathway would reveal the exact mechanism behind teratogenesis, resulting from apoptosis induced compensatory proliferation (CP) of the embryonic cells under the influence of hypoxia.

### Conclusion: p53 and its role in malformation

It can be therefore concluded from this study that hypoxia during embryonic development in gold fish, leads to malformation, owing to disruption of the PCD infrastructure. More precisely, this malformation is p53 dependent. Here, we have found reverse abundance of p53 in hypoxic embryos during 24 hpf; during initial 12 hpf, its upregulation caused cell cycle arrest and thereafter, its downregulation resulted in uncontrolled cell proliferation. p53 is mostly required in fish larval development after 10–12 hpf, when segmentation of body parts take place. Simultaneously, caspase-3 activated programmed cell death maintains normal differentiation pattern [35]. It has been observed that *Xenopus* embryos, treated with p53 inhibitor, blocked the ability of early blastomeres to undergo differentiation and resulted in tumor formation, whereas, further injection with wild type-p53 resulted in suppression of tumor [36]. Here, hypoxia played a limiting role by downregulating p53 and caspase-3 almost simultaneously after 12 hpf, which further lead to incapability in arresting cell proliferation. This probably happened due to paucity of p53 dependent neutralization of anti-apoptotic Bcl-2 protein, in spite of upregulation of Bcl-2 as a whole. So, the phenomenon of compensatory proliferation became disastrous for embryos in the present study, as p53 was downregulated during post 12 hpf period. Therefore, timely activation of p53 is one of the key factors determining or resulting in normal development/differentiation, but, hypoxia disrupts the p53 clock/machinery, thereby causing malformation, through failure of the PCD infrastructure.
